# Multifaceted Role of Microbiota‐Derived Indole‐3‐Acetic Acid in Human Diseases and Its Potential Clinical Application

**DOI:** 10.1096/fj.202500295R

**Published:** 2025-05-26

**Authors:** Nargis Shaheen, Jiaxing Miao, Boyu Xia, Yutong Zhao, Jing Zhao

**Affiliations:** ^1^ Department of Physiology and Cell Biology, College of Medicine The Ohio State University Columbus Ohio USA; ^2^ Davis Heart and Lung Research Institute The Ohio State University Columbus Ohio USA

**Keywords:** gut microbiota, human diseases, indole‐3‐acetic acid, pathogenesis, therapeutic strategy

## Abstract

Indole‐3‐acetic acid (IAA) is recognized as a common plant growth hormone. It is also detectable in mammals, as it can be produced by gut microbiota in the gut. Current research works have shown that IAA is a multifaceted compound with significant consequences for several physiological and pathological processes. Higher levels of IAA occur in chronic kidney disease because decreased renal clearance contributes to cardiovascular and liver dysfunction. In contrast, IAA demonstrates protective effects by modulating oxidative stress, inflammation, and lipid metabolism in nonalcoholic fatty liver disease. IAA also shows potential therapeutic benefits in lung disorders such as acute lung injury, pulmonary fibrosis, and chronic obstructive pulmonary disease. Furthermore, IAA shows potential as an adjunct in cancer therapy, where it synergizes with chemotherapy regimens by endorsing oxidative stress and autophagy. Beyond cancer‐related applications and metabolic disorders, IAA's role in neuroinflammation, mainly in conditions such as sepsis‐associated encephalopathy and depression, underscores its potential as a therapeutic agent targeting the gut–brain axis. Furthermore, IAA is a potent oral hypoglycemic agent with mitigating effects on metabolic disorders associated with obesity and diabetes. This review provides an overview of the diverse roles of IAA in disease pathophysiology, its therapeutic promise, and the need for further research to better understand its mechanistic pathways. Understanding these complex effects could pave the way for novel microbiota‐based therapies to address a range of chronic diseases and improve patient outcomes.

## Graphical abstract

The complexity of indole‐3‐acetic acid's (IAA's) role in human health and disease. IAA is a multifaceted compound with significant consequences for several physiological and pathological processes.

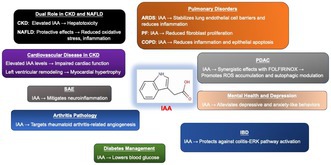

## 1 Introduction

Intestinal dysbiosis is characterized by an imbalance in microbiota, and it is linked to inflammation, oxidative stress, and various diseases, such as metabolic disorders and inflammatory bowel disease (IBD) [1, 2, 3]. The fecal microbiota transplantation (FMT) has emerged as an auspicious therapy for dysbiosis‐associated diseases [1, 4, 5, 6]. Restoring gut microbiota homeostasis is seen as a potential means to alleviate these conditions, as studies have shown that transplanting microbiota from individuals with inflammation to germ‐free mice increases susceptibility to colitis [7].

**FIGURE 1:**
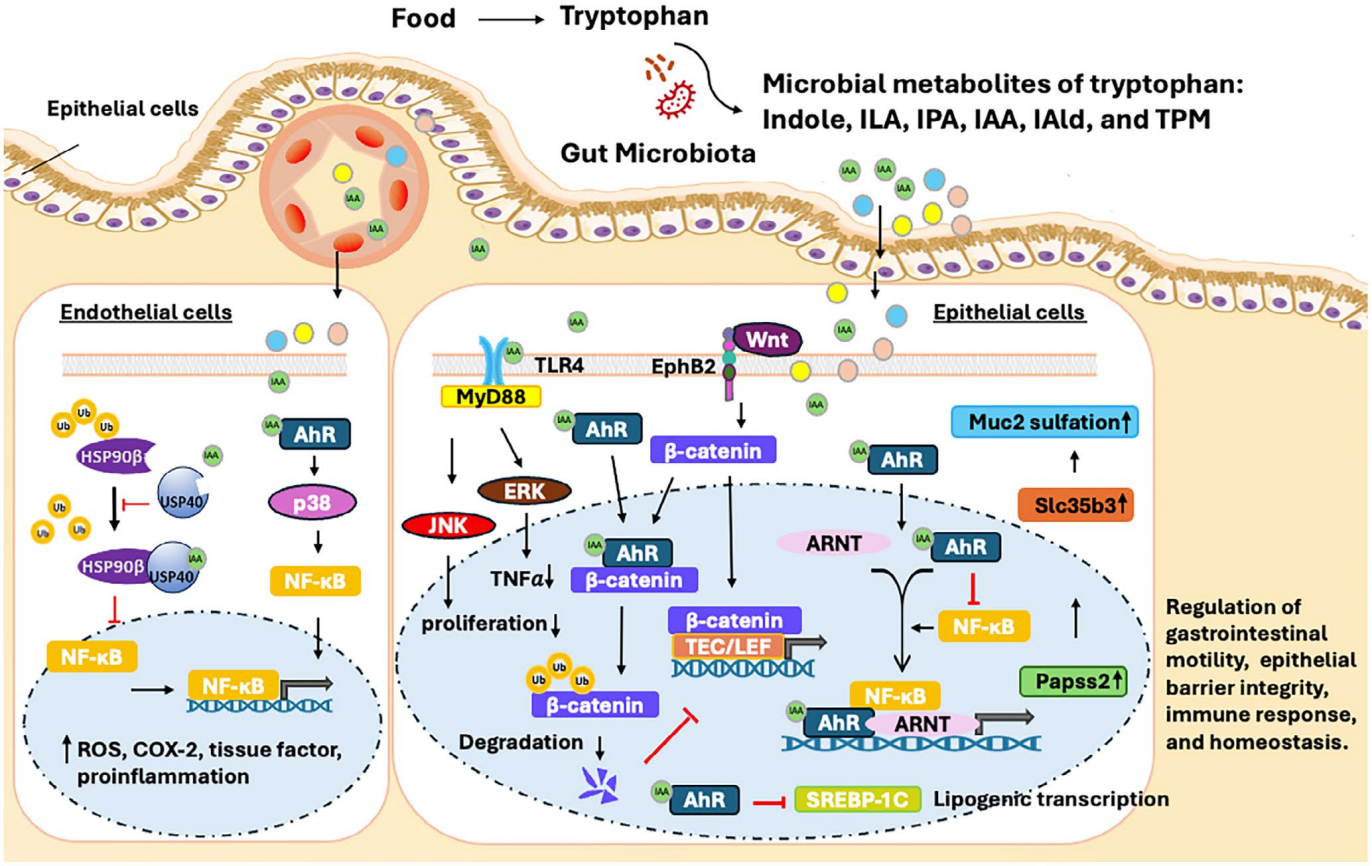
Indole‐3‐acetic acid (IAA)‐mediated signaling pathways in epithelial and endothelial cells. In endothelial cells: IAA acting as a USP40 activator stabilizes the endothelial barrier integrity and reduces inflammation by enhancing HSP90β deubiquitination, consequently inhibiting NF‐kB activity. Elevated IAA levels in patients with chronic kidney disease (CKD) induce endothelial oxidative stress and inflammation by increasing reactive oxygen species (ROS) and expression of COX‐2 and tissue factor via activating the AhR/p38MAPK/NF‐kB pathway, which contributes to the high cardiovascular risk of patients with CKD. In epithelial cells: IAA may act as a TLR4 ligand ameliorates colitis by activation of ERK signaling and inhibits cell proliferation through TLR4‐JNK activation. The crosstalk between IAA‐AhR and Wnt/β‐catenin promotes intestinal stem cells (ISCs) homeostasis. IAA activates AhR can improve gut motility and enhance intestinal mucin sulfation through the AhR‐3′‐phosphoadenosine 5′‐phosphosulfate synthase 2 (Papss2)‐solute carrier family 35 member B3 (Slc35b3) pathway, contributing to the protection of intestinal homeostasis. IAA modulates lipid metabolism via the AhR/SREBP‐1C pathway in liver. Indole‐3‐acetonitrile (IAN), the indole‐3‐acetamide (IAM), the tryptophan side‐chain oxidase (TSO), the tryptamine (TPM), and the indole‐3‐pyruvic acid (IPA/IPyA), indole 3 aldehyde (IAld), heat shock protein 90 (HSP90), Ubiquitin carboxyl terminal hydrolase 40 (USP40), aryl hydrocarbon receptor (AhR), aryl hydrocarbon receptor nuclear translocator (ARNT), sterol regulatory element binding protein‐1c (SREBP‐1c), T cell factor (TCF)/lymphoid enhancer binding factor (LEF), Ephrin type B receptor 2 (EphB2).

The microbial metabolites play an important role in the daily nutrient intake, the gut microbiome, and host health [8, 9]. Research studies increasingly highlight the impact of gut microbiota‐generated metabolites on maintaining immune system balance and supporting gastrointestinal health [10]. Among these metabolites, tryptophan‐derived microbial metabolites, such as indole‐3‐acetic acid (IAA), are particularly important due to their contributions to gastrointestinal function, anti‐inflammatory effects, antioxidant properties, and immune modulation [11, 12, 13, 14]. IAA is especially beneficial for intestinal health through activating the aryl hydrocarbon receptor (AhR), which supports the integrity of gut epithelial cells, maintains the intestinal barrier, and has anti‐inflammatory effects in the gastrointestinal tract [14, 15, 16]. The beneficial effects of IAA are not limited to the intestine but also influence the central nervous system (CNS) and metabolism. The major signaling pathways involved in IAA‐mediated intestinal homeostasis, barrier integrity, immune response, and lipid metabolism through activation of AhR signaling or AhR‐independent signaling are summarized in Figure 1. This makes IAA a promising candidate for managing gastrointestinal and neurological disorders [5, 11, 16, 17]. However, although IAA is a small, fat‐soluble molecule that can be readily absorbed in the small intestine [16], delivering it competently to the colon still remains challenging. Oral administration often requires high doses (e.g., 50 mM in drinking water); thus, this drug delivery route may not be suitable for clinical use [15].

Key bacterial genera have been identified as contributors to the synthesis of IAA from tryptophan, highlighting their specific roles, mechanisms, and relevant references (Table 1). Bacterial cross‐feeding on intermediate metabolites like indole lactic acid facilitates cooperative IAA production among gut microbiota. The enzymes involved vary among species, with pathways such as the arylformamidase pathway and the tryptophanase‐catalyzed pathway being central to IAA synthesis. In this review, we will summarize the recent studies regarding IAA biosynthesis, its roles in the development of human diseases, and its potential in treating human diseases.

**TABLE 1:** Bacteria involved in IAA synthesis in mammal's microbiome.

Bacteria	Role in IAA synthesis	Mechanisms	Functions
*Clostridium*	Metabolizes ILA to produce IAA and IPA through induced tryptophan‐metabolizing enzymes [5]	Converts tryptophan to IAA via the arylformamidase pathway involving enzymes like indoleacetamide hydrolase	Attenuates the MCT‐induced liver injury [18]
*Roseburia*	Enriched in response to ILA, contributing to IAA production [19, 20]	Uses enzymes like tryptophanase to convert tryptophan to indole, which is further processed to IAA	In treatment of IBD and metabolic diseases/conditions [20]
*Faecalibacterium*	Increased in abundance with ILA treatment, aiding IAA synthesis [21]	Converts tryptophan to indole derivatives, processed further to form IAA	Alleviates symptoms of Crohn's disease, enhance the intestinal barrier function and DSS‐induced colitis [1, 21, 22]
*Lactobacillus (L. reuteri)*	Produces ILA, which induces IAA synthesis in the gut microbiota [5, 11]	ArAT enzyme catalyzes tryptophan to ILA, which is further metabolized by other bacteria to IAA	Alleviates intestinal inflammation, MCT‐induced liver injury, protective effects in COPD mouse model, liver steatosis, obesity in mice, HFD‐induced colitis, endotoxemia, and anti‐depression‐like behavior [5, 11, 18, 20, 23, 24, 25]
*Bifidobacterium*	Associated with higher IPA and IAA levels in certain populations [26]	Contributes to tryptophan metabolism, enhancing the production of IAA and IPA	Alleviates MCT‐induced liver injury, liver steatosis, obesity, HFD‐/DSS‐induced colitis, asthma airway inflammation, metabolic disorder [18, 24, 27, 28, 29]
*Bacteroides fragilis, Bacteroides thetaiotaomicron*	Producing IAA by metabolizing tryptophan [26]	ArAT enzyme catalyzes tryptophan to PLA, which is further metabolized by phenyllactate dehydrogenase to IAA	Alleviates MCT‐induced liver injury and DSS‐induced colitis [18, 22]

## 2 Biosynthetic Pathways of IAA in Microorganisms

**FIGURE 2:**
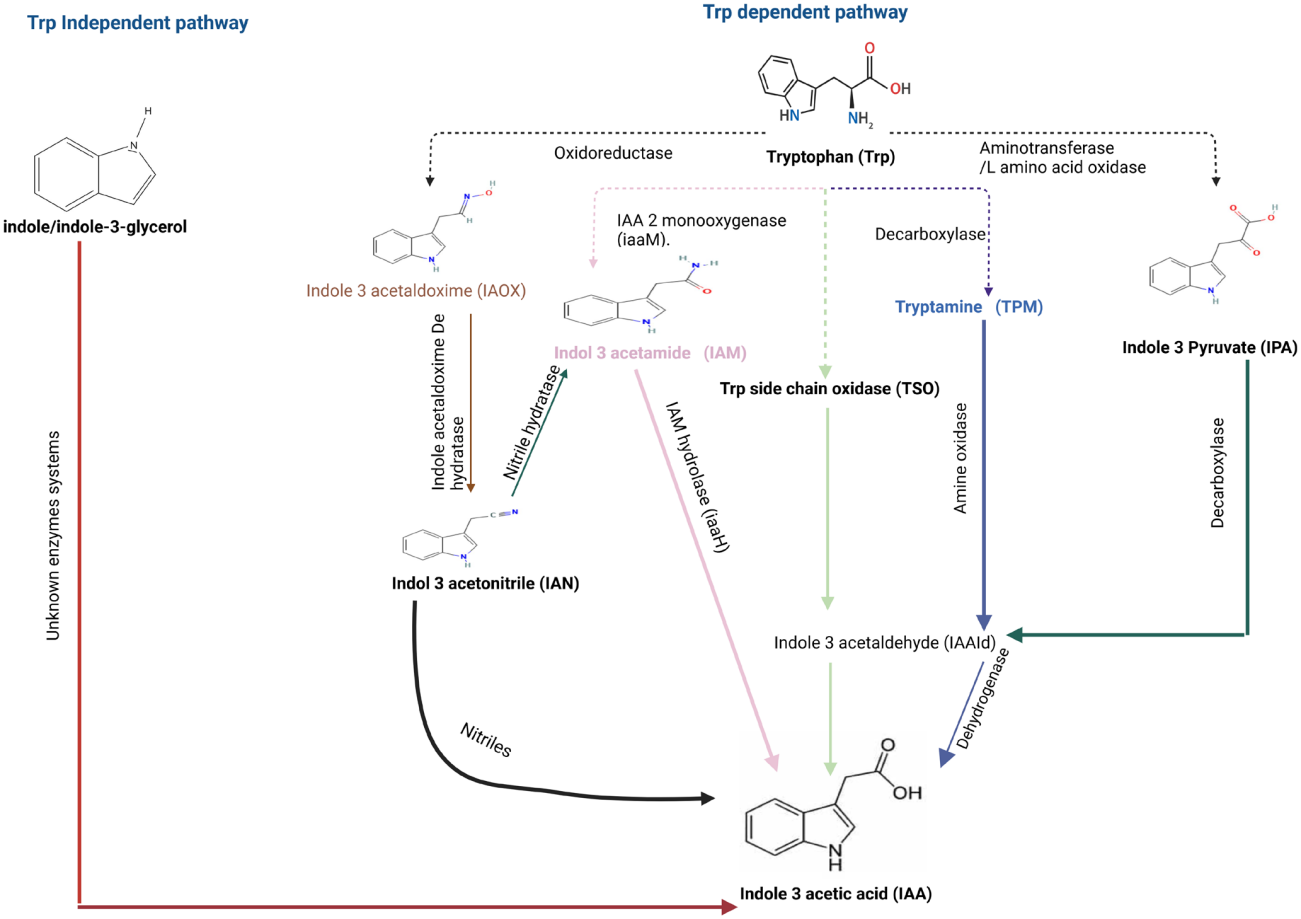
Biosynthetic pathways of IAA in microorganisms. The main microbial IAA synthesis pathways. Various pathways are depicted with different colors. Trp‐independent pathway (the red arrow on the left): IAA biosynthesis from indole/indole‐3‐glycerol, tryptophan‐independent IAA synthesis pathway with unclear enzyme systems. Trp‐independent pathway (on the right): The first part of the five major tryptophan‐dependent pathways showed tryptophan‐catalyzing step (dash lines). The second part of tryptophan‐dependent pathways showed IAA synthesis from intermediates (IAN, IAM, TSO, TPM, and IPA). The figure was created with biorender.com.

The biosynthesis of IAA in microorganisms can be categorized into two main pathways: tryptophan (Trp)‐dependent and tryptophan‐independent. The classification is based on Trp serving as a precursor in the synthesis process. These microbial pathways bear some resemblance to the mechanisms by which plants produce IAA [30]. In microorganisms, tryptophan‐dependent pathways play a key role in IAA biosynthesis through five primary pathways named after the key intermediate metabolites of tryptophan: the indole‐3‐acetonitrile, the indole‐3‐acetamide (IAM), the tryptophan side‐chain oxidase, the tryptamine, and the indole‐3‐pyruvic acid (IPA) [31]. L‐tryptophan, the central precursor in the synthesis of IAA within these pathways, is a rare amino acid with a high energy cost for biosynthesis, making it less abundant in microbial cells. As a result, microorganisms typically require an excess of external tryptophan to produce significant levels of IAA. Among these pathways, the IAM and IPA pathways are the most widely observed IAA biosynthetic routes in microorganisms [32]. Additionally, a few microorganisms are known to synthesize IAA via tryptophan‐independent pathways that rely on indole‐3‐glycerol phosphate or indole as precursors [33, 34]. Nevertheless, these alternative pathways are less understood, with key enzymes and genes involved yet to be identified in depth (Figure 2). IAA serves not only as a signaling molecule that regulates physiological processes and microbial adaptation to external stresses but also plays a crucial role in microorganism–plant interactions [30, 32]. Therefore, IAA influences plant growth, development, and even physiological and pathological responses. Thus, understanding microbial IAA biosynthesis routes is valuable for discovering the guideline of IAA synthesis in microorganisms and its functional roles in microbial and plant biology [31, 32].

## 3 Accumulation and Impact of IAA in Chronic Kidney Disease

The chronic kidney disease (CKD) poses a significant global health threat, impacting between 11.7% and 15.1% of the global population. Approximately 4.9–7.1 million people worldwide require renal replacement therapy due to end‐stage kidney disease [35]. In CKD patients, the inability of the kidneys to effectively eliminate organic compounds leads to uremic syndrome. This syndrome is primarily driven by an accumulation of substances, discussed as uremic toxins when they display biological activity, that the kidneys would typically process or eliminate [36]. These uremic toxins detrimentally affect nearly every organ system, with the cardiovascular (CV) system being particularly vulnerable [37].

IAA is present in certain ready‐to‐eat foods due to its widespread use and has become easily available because of natural and synthetic sources for overuse [38]. Due to its strong binding affinity with albumin, IAA demonstrates reduced excretion rates and is only partially removed during hemodialysis [39]. This implies that decreased renal function may contribute to IAA accumulation in the body.

Exposure to elevated levels of IAA in the environment may pose health risks to individuals with CKD, as reported by Gondouin et al. [40]. Although multiple association studies and in vitro experiments have investigated IAA's effects, it has yet to be definitively identified as an autonomous risk factor for CKD. p‐cresyl sulfate and indoxyl sulfate are two well‐known uremic toxins with nephrovascular toxicity [41]. A study by Alhusaini et al. demonstrated the renoprotective effects of IAA in valproate‐induced kidney injury [42]. IAA administration did not exhibit nephrotoxic properties. These findings are supported by the similar levels of serum phosphorus and creatinine. Additionally, histological analysis revealed no changes in mice kidney morphology exposed to IAA, although additional molecular investigations are necessary to confirm these conclusions [43].

## 4 Impact of IAA on CV Health in CKD

The cardiac hypertrophy is considered the heart's compensatory response to increased demands for blood circulation. The reduced ejection fraction and left ventricular remodeling observed in the CKD group may be attributed to renal dysfunction, which can cause fluid buildup and place additional strain on the heart [44]. Left ventricular hypertrophy is a CV complication observed in individuals with CKD or ESRD. It is characterized by an increase in the size of the left ventricle of the heart and metabolic disturbances associated with kidney dysfunction [45, 46]. This pathological condition presents a significant treatment challenge, meaningfully contributing to the elevated CV morbidity and mortality seen in those affected by CKD.

Research studies including in vitro and epidemiological studies have linked IAA to various CKD‐associated complications [47, 48]. With normal kidney function, IAA is efficiently excreted via urine, as shown in the IAA‐only group [49]. Analyzing serum and urine samples through high‐performance liquid chromatography (HPLC) revealed that CKD + IAA groups had higher serum IAA and lower urinary IAA excretion [43]. Previous studies offer empirical support that IAA contributes to CV dysfunction in the adenine‐induced CKD rat model or the patients with CKD [43, 47]. The CKD + IAA group exhibited myocardial hypertrophy, reduced cardiac ejection fraction, and worsened CV function with left ventricular remodeling compared to the CKD‐only group. Nayak et al. revealed a marked increase in cardiac biomarkers, including C‐troponin, Creatine kinase isoenzyme MB (CK‐MB), and Lactate Dehydrogenase, in the CKD + IAA group, underscoring the cardiotoxic impact of IAA, while the CKD group exhibited no significant increase. Histological analysis of heart tissue also demonstrated more pronounced morphological changes in the CKD + IAA group compared to other experimental groups [43]. The beta‐myosin heavy chain (β‐MHC), *B*‐type natriuretic *peptide* (BNP), and atrial natriuretic peptide (ANP) are known indicators of heart failure and myocardial infarction, making them critical in cardiac health assessment [50]. The study by Nayak et al. demonstrated an increase in hypertrophic markers, such as ANP, BNP, and β‐MHC, in CKD groups treated with IAA, suggesting that IAA plays a role in the cardiac hypertrophy development [43]. The study group further elucidated the mechanism underlying IAA‐induced cardiotoxicity in Wistar albino rats. The study mimicked the circulating IAA concentration previously reported in CKD patients. IAA‐treated animals displayed decreased superoxide dismutase (SOD) and catalase levels, and elevated lipid peroxidation and inflammation response in heart tissue. IAA treatment induced significant cardiac alterations, including increased heart size, myocardial thickening, and hypertrophy [51]. Moreover, higher cardiac fibrosis markers, including Col‐I and Col‐III, were observed in the CKD rat or Wistar albino rats treated with IAA [43, 51]. By activating the AhR pathway, IAA induces endothelial oxidative stress and inflammation by increasing ROS and expression of COX‐2 and tissue factor via activating the AhR/p38MAPK/NF‐KB pathway, which contributes to the high CV risk of patients with CKD [47, 52].

## 5 Impact of IAA on Liver Health in CKD

The liver is a vital organ involved in numerous physiological functions, including immune responses and metabolism, detoxification. It plays a critical role in hormone production and regulation of various proteins [53]. The study conducted by Nayak et al. suggested that CKD rats exposed to IAA displayed increased liver damage, as evidenced by elevated levels of alanine transaminase (ALT), aspartate transferase (AST), alkaline phosphatase, and total bilirubin in the blood [43, 54]. These findings align with prior research suggesting that excessive IAA exposure may lead to hepatic stress [55]. In this case, liver stress and damage were linked to reduced kidney filtration and elevated blood levels of IAA because of CKD. Histological examination of liver tissues from the IAA‐exposed CKD group revealed specific alterations, including localized cell necrosis, sinusoid congestion, cytoplasmic vacuolization, hydropic degeneration, and cellular infiltration. These observations suggest that IAA presence in CKD leads to changes in liver structure and impairs liver function [43, 54]. Given the liver's vital role in lipid metabolism, the CKD group exposed to IAA also exhibited dyslipidemia, a known risk factor for CV disease [43].

## 6 Role of IAA in Inflammatory Bowel Disease

The IBD is a chronic inflammatory disorder affecting the gastrointestinal tract, with a globally increasing prevalence in recent decades, although its exact etiology remains unclear [56]. Despite significant dietary and genetic differences among IBD patients from various regions, a consistent pattern of gut microbiota alterations has been observed. IBD patients exhibit a significant reduction in commensal gut microbiota, which play critical roles in host health and function [57].

A western high‐fat diet (HFD) has been recognized as a risk factor for IBD [58]. A study reported by Li et al. showed a positive correlation between dietary fat intake and disease severity in both murine colitis models and IBD patients [11]. A HFD was shown to significantly reduce IAA levels, leading to intestinal barrier damage. In contrast, supplementation with IAA enhances intestinal mucin sulfation and effectively alleviates colitis. Mechanistically, IAA upregulates key molecules involved in mucin sulfation, including the AhR‐3'‐phosphoadenosine 5'‐phosphosulfate synthase 2 (Papss2) and solute carrier family 35 member B3, the enzyme and transferase responsible for synthesizing 3'‐phosphoadenosine‐5'‐phosphosulfate (PAPS) via activation of AhR. Importantly, AhR directly binds to the transcription start site of Papss2 [11]. Furthermore, oral administration of *Lactobacillus reuteri* , a bacterium capable of producing IAA, has been shown to protect against colitis and promote mucin sulfation. However, a modified strain of *L. reuteri* lacking the iaaM gene (*Lactobacillus* ΔiaaM), and thereby its ability to produce IAA, failed to demonstrate these protective effects [11].

IAA possess the ability to alleviate intestinal inflammation of the intestine and modulate the gut microbiota in both IL‐10^−/−^ spontaneous and DSS‐induced colitis models [5]. Additionally, Qu et al. reported that supplementation with IAA showed protective effects against the DSS‐induced colitis mouse model by reducing weight loss, disease activity, and colon injury while improving intestinal barrier function. IAA activated the ERK signaling pathway; blocking ERK with U0126 reduced IAA's benefits, highlighting its impact in colitis protection of colitis [15].

## 7 Therapeutic Potential for Nonalcoholic Fatty Liver Disease

Nonalcoholic fatty liver disease (NAFLD), the most common chronic liver disease globally, is closely linked to obesity, insulin resistance, and dyslipidemia. NAFLD affects approximately 24% of the global population, with 5%–20% progressing to nonalcoholic steatohepatitis [59]. NAFLD is closely linked to gut microbiota‐derived metabolites (MDTMs). Ji et al. reported that IAA has been shown to mitigate the severity of hepatotoxicity in HFD‐fed mice. IAA administration improved HFD‐induced insulin resistance, dyslipidemia, oxidative stress, and inflammation, thus demonstrating its potential as a therapeutic agent for NAFLD [60]. Mice fed with HFD exhibited weight gain, insulin resistance, hyperglycemia, and dyslipidemia, although IAA treatment improved all these conditions except weight gain, likely due to its function as an AhR ligand [60, 61, 62]. Similarly, FICZ, another AhR ligand, has been reported to improve insulin resistance and lipid metabolism without affecting body weight in HFD‐fed mice [61]. IAA treatment alleviates HFD‐induced liver injury as shown by the reduction in macrophage infiltration and the expression of TNF‐α and MCP‐1 in hepatic tissue. Elevated serum glutamic‐pyruvic transaminase or ALT levels, an indicator of liver damage, were reduced by IAA treatment, confirming its protective effect against liver injury [60, 62]. IAA treatment significantly reduced the total triglycerides and cholesterol levels in the liver of mice fed with HFD, which may be associated with the negative regulation of gene expression related to lipogenesis, including *Srebf1*, *Scd1*, *PPARγ*, *Acaca*, and *Gpam* [60, 62]. Oxidative stress plays a central role in NAFLD progression by inducing lipid peroxidation, insulin resistance, lipid accumulation, and inflammation [60, 62]. HFD‐induced suppression of antioxidant defense systems, including reduced SOD activity and glutathione levels, was reversed by IAA [60]. IAA treatment reduced hepatic ROS, lipid peroxidation product malondialdehyde levels, and macrophage infiltration, potentially due to its free radical scavenging ability [13, 60]. Overall, these findings are in accordance with findings reported by Ma et al. The circulating levels of indole were reversely correlated with body mass index. Indole treatment significantly decreases in the severity of hepatic steatosis and inflammation in HFD‐fed C57BL/6J mice in a myeloid cell PFKFB3‐dependent manner [63]. Furthermore, Xu et al. demonstrated that IAA modulated liver lipid metabolism through activation of the AhR/SREBP‐1C signaling pathway [64]. Briefly, IAA alleviates hepatic steatosis, oxidative stress, and inflammation in NAFLD, highlighting its potential as a therapeutic agent. These findings contribute to understanding the protective role of gut MDTMs in NAFLD progression. IAA alleviates hepatotoxicity caused by a diet consisting of high fat, contributing to enhanced insulin sensitivity, better lipid metabolism, and decreased oxidative and inflammatory stress. Incorporating tryptophan‐rich foods, which support the production of IAA by gut microbiota, may aid in preventing liver‐related pathological conditions [65].

As discussed above, IAA has opposite roles in different liver conditions. In contrast to the previously reported anti‐inflammatory agent in mice, IAA markedly amplified the inflammatory response and cellular damage in D‐GalN/LPS‐induced acute liver failure. IAA pretreatment aggravates the acute injury in the liver with elevated ALT and AST levels. Additionally, toll‐like receptor 2 (Tlr2) expression was significantly upregulated in the liver of IAA‐pretreated mice challenged with D‐GalN/LPS [66]. The role of TLR2 in infectious diseases is still controversial [67]. However, hyper‐inflammation caused by excessive TLR2 signaling can lead to tissue damage and wound healing impairment [68]. Oral gavage of IAA may aggravate the liver injury via the upregulation of the TLR2/NF‐κB signaling pathway [66]. Future research should consider the potential effects of IAA more carefully, for example, the routes for administration which may have diverse impact especially on gut microbiota composition.

## 8 Effects of IAA on Diabetes and Obesity

Research dating back to 1956 demonstrated that single doses of IAA (100 mg/kg body weight) are non‐toxic and can effectively lower blood glucose levels in diabetic patients [69]. The findings were based on the observation of the acute effects of IAA by mouth on the blood sugar concentration of a small number of diabetic patients. The mechanism underlying IAA‐decreased blood sugar in adult patients with diabetes mellitus and the long‐term toxic effects of IAA remain unclear.

Obesity is a metabolic disorder which is characterized by a chronic low‐grade inflammation that highly affects immune and metabolic functions [70]. Su et al. used an obesity mice models and they observed that the gut Reg4 promoted IAA (500 mg/kg)‐induced generation of IL‐35^+^ B cells in the presence of LPS (2 mg/kg). They also observed that the level of IAA was lower in humans with obesity as compared to non‐obese subjects [71]. These findings may support the idea that IAA‐producing *Lactobacilli* may play a protective role against obesity. Oluwagbemigun et al. examined long‐term body mass index (BMI) patterns related to urinary amino acids and their link to C‐reactive protein (CRP) levels in late adolescence and young adulthood. They observed that males with persistent overweight had lower levels of IAA and elevated CRP. Although these findings were restricted to males only [72], they are potentially important and provide evidence that IAA or modification of the intestinal microbiota could be therapeutic for the amelioration of inflammation and metabolic disorders in obese individuals.

## 9 IAA as a Potential Therapeutic for Acute Respiratory Distress Syndrome

Acute respiratory distress syndrome (ARDS) is a serious lung dysfunction condition that is characterized by edema caused by loss of lung microvascular endothelial cell (HLMVEC) integrity [73]. A recent study from our group investigated the effects of IAA on lung EC inflammation and permeability. IAA pretreatment protected against bacterial endotoxin (LPS)‐induced HLMVEC barrier disruption. Furthermore, IAA inhibited an anti‐inflammatory effect in HLMVECs. In an LPS‐induced ALI murine model, IAA pretreatment decreased pulmonary microvascular leakage, reduced immune cell infiltration, and lowered cytokine levels in bronchoalveolar lavage, significant protective effects against lung injury. Mechanically, IAA increased the deubiquitinase activity of USP40, which reduced the ubiquitination of HSP90β, stabilizing the EC barrier and decreasing inflammation. IAA also enhanced the association between USP40 and HSP90β. In EC‐specific USP40 deficient mice, IAA failed to protect against LPS induced lung injury, confirming USP40's critical role in mediating IAA's protective effects [12]. In this study, IAA was intraperitoneally injected into mice, and we did not investigate the role of the gut microbiota‐generated IAA in lung injury. Gut dysbiosis‐caused gut‐lung crosstalk has been shown to promote lung injury [74]. It is unclear whether the gut IAA affects lung ECs in the setting of ARDS. The therapeutic potential of IAA in ARDS needs more preclinical studies, such as posttreatment of IAA in preclinical models of ALI and intratracheal administration of IAA.

## 10 IAA as a Potential Therapeutic for Pulmonary Fibrosis

Pulmonary fibrosis (PF) is a chronic lung disease categorized by scarring and thickening of lung tissues. Uncontrolled and repeated lung injury and repair and remodeling cause activation of fibrotic fibroblasts and extracellular matrix accumulation [75]. There is a significant reduction in IAA level in both the patients of PF and experimental PF mouse models. There are three perspectives regarding the underlying mechanisms of IAA's antifibrotic effects. First, IAA enabled autophagy recovery in TGF‐induced fibroblasts by PI3K/AKT/mTOR pathway inhibition, thereby reducing fibroblast migration and proliferation. Secondly, it reduced senescence in alveolar epithelial cells through the modulation of PI3K/AKT and Hif‐1 signaling. Finally, IAA altered lung microbiota composition and structure in the BLM‐induced PF mouse model. Together, these observations state that IAA has a potential for PF treatment. In this study, the authors administered IAA after 7 days of bleomycin challenge; the strategy mimics therapeutic treatment. The bleomycin model is a widely used animal model of PF; however, PF caused by bleomycin is reversible. Thus, a repeated bleomycin challenge‐induced PF model or bleomycin‐challenged aging mice may be used to investigate the effect of IAA on treating PF [76].

## 11 IAA as a Potential Therapeutic for Chronic Obstructive Pulmonary Disease

Chronic obstructive pulmonary disease (COPD) is a chronic and progressive lung disorder characterized by airway inflammation and excess mucus production. Previous studies have observed that patients with COPD and hepatic steatosis tend to have reduced levels of IAA. Additionally, IAA supplementation has shown promise in enhancing lung function, decreasing cell death, and reducing inflammation in COPD [23]. Neutrophilic COPD exhibits a stronger association between microbial and host pathways, with disrupted amino acid metabolism, specifically reduced tryptophan catabolism, leading to decreased production of IAA. This reduction contributes to heightened inflammation, epithelial apoptosis, and lung function decline, revealing IAA as a potential protective metabolite in COPD. A study by Gao et al. characterized the airway microbiome and human genetics. Deep metagenomic sequencing in the sputum of 99 COPD and 36 healthy individuals identified correlations between the expression of host genes and their genetically linked microbiome features [2]. Furthermore, a sequential mediation analysis of multi‐omics data from the cohort individuals revealed the microbiome–metabolite–host interaction. *Lactobacilli*, including *L. salivarius* , a potential IAA producers in the airways, have been used to prevent and treat gastrointestinal disorders [77]. Their presence in airway microbiomes suggests a role for oral commensals in lung health [78, 79]. Altered tryptophan metabolism in airway lactobacilli associated with reduced IAA was observed in neutrophil‐predominant COPD, which was in turn linked to perturbed host interleukin‐22 (IL‐22) signaling. Airway microbiome‐derived IAA mitigates COPD pathophysiology by reducing neutrophilic inflammation, epithelial apoptosis, emphysema, and lung function decline, via macrophage–epithelial cell crosstalk mediated by IL‐22 signaling [23]. Moreover, intranasal inoculation of two airway *lactobacilli* restored IAA and recapitulated its protective effects in mice, which provided a novel therapeutic approach in targeting microbe–host interaction in COPD [23]. A study by Jayaraman et al. demonstrated that IAA has inhibitory effects on biofilm formation and virulence production of *Pseudomonas aeruginosa* [80]. Differences in transcriptional activity between microbial species could explain the observed decrease in IAA in COPD airways, warranting further investigation. Other indole derivatives, including indole‐3‐lactic acid, indole‐3‐propionic acid, and indole‐3‐acetaldehyde, are known mediators of microbe–host communication and may also play roles in COPD pathogenesis. Understanding their contributions could further refine therapeutic approaches.

## 12 IAA in Tumorigenesis and its Potential Neoadjuvant for Tumor Therapies

The gut microbiota can produce a variety of microbial‐derived metabolites to influence tumor development [81]. Intestinal homeostasis and regeneration are driven by the normal turnover of intestinal stem cells (ISCs) lying in the intestinal crypt. The microbiota, especially crypt‐specific microbiota, continuously stimulate ISCs proliferation and differentiation to repair the intestinal epithelium to maintain the integrity of the mucosal barrier. Zhang et al. revealed that *Acinetobacter radioresistens* (a member of the crypt specific microbiota) plays a key role in inhibiting ISCs proliferation and turnover through IAA production [82]. The AhR signaling in response to IAA and its crosstalk with the Wnt/β‐catenin signaling pathway are essential for *A. radioresistens* ‐mediated effects on ISC proliferation and tumorigenesis. *A. radioresistens* ‐derived outer membrane vesicles (OMVs) inhibit cell proliferation in human colonoids in an IAA‐dependent manner. *A. radioresistens* ‐derived IAA inhibits both tumor initiation and spheroid formation in APC^Min/+^ mice (a Wnt‐driven mouse model of intestinal adenomas) along with tumor cell proliferation in colonic tumor slice cultures derived from colorectal cancer (CRC) patients. Furthermore, specific clearance of *A. radioresistens* using the lytic bacteriophage (Ar‐Ф) shows that *A. radioresistens* promotes intestinal epithelial homeostasis under normal conditions by regulating the Wnt/β‐catenin pathway. Together, the protective effects of *A. radioresistens* are mediated by the IAA‐AhR‐Wnt‐β‐catenin signaling axis in crypts. Gastrointestinal MDTMs have emerged as critical regulators of host health and homeostasis. The study by Zhang et al. highlights an *A. radioresistens* –ISC crosstalk that influences colonic turnover and the potential of OMVs as a delivery tool for IAA in the treatment of CRC, especially in CRC patients with a specific decline in *A. radioresistens* .

Chemotherapy remains the primary treatment option for metastatic pancreatic ductal adenocarcinoma (PDAC), despite a response rate of less than 50% to initial therapies [83]. In shotgun metagenomics and metabolomics screening, Tintelnot et al. revealed that IAA increases chemotherapy efficacy in PDAC patients. In humanized gnotobiotic mouse models of PDAC, dietary manipulation of tryptophan, FMT, and oral IAA increase treatment response. The myeloperoxidase‐driven oxidation of IAA increases ROS accumulation and downregulation of autophagy, which impairs cancer cell metabolism and ultimately proliferation. In PDAC patients, they observed a strong correlation between the concentration of IAA and therapy efficacy, highlighting its potential for nutritional interventions in cancer treatment [84].

Building on the concept of elevated IAA levels in the plasma of cancer patients, which has been associated with improved survival outcomes [84], Vaaben et al. engineered *Escherichia coli* Nissle 1917 to produce IAA. In a murine tumor model, the administration of this engineered bacterial strain changed the tumor microenvironment by elevating IFN‐γ and CXCL9 levels, increasing T‐cell infiltration, and enhancing immune activation in the murine tumor model. The treatment significantly suppresses tumor progression, prolongs survival in syngeneic tumor models, and leads to long‐lasting anti‐tumor immunity in rechallenge experiments. Their investigations also stated that this immune alteration is driven by the direct AhR activation, which leads to increased cytokine expression and a shift in the immune cell composition within the tumor [85]. These observations underscore the potential of microbial metabolites in immune modulation and support further investigations into microbiome‐based cancer immunotherapies.

## 13 IAA for Depression

Depression is a multifaceted mental health condition, affecting approximately 10% of the global population. It is associated with various forms of brain dysfunction, including cognitive impairment, memory issues, and a diminished ability to experience pleasure (anhedonia) [86]. Current antidepressants, such as selective serotonin reuptake inhibitors and monoamine oxidase inhibitors, function by increasing neurotransmitter levels. However, their use often comes with notable side effects, driving research toward alternative treatment options [87].

Emerging evidence highlights the significant role of gut microbiota in mitigating depression. The gut microbiota influences essential physiological processes, including immune regulation, brain activity, and bacterial metabolism [88]. Psychobiotics, primarily derived from bacterial strains like *Lactobacillus* and *Bifidobacterium*, have shown promise in reducing anxiety and depression symptoms [89]. These effects are achieved by modulating gut microbiota composition in both animal models and human patients.

Tian et al. reported that the IAA content fluctuates in the cerebrospinal fluid, urine, and feces of patients with depression [90]. Recent experiments in a chronic mild stress model in mice demonstrated that IAA supplementation with 50 mg/kg over 5 weeks reduced depressive and anxiety‐like behaviors [17]. This treatment also improved hypothalamic–pituitary–adrenal axis function and boosted levels of brain‐derived neurotrophic factors. Moreover, IAA helped restore microbial metabolite balance in the colon but did not directly affect indole concentrations in the brain [17]. These findings suggest that IAA exerts antidepressant effects potentially through the modulation of gut microbiota and its metabolites. However, further research is needed to elucidate the molecular mechanisms underlying their impact on the CNS and emotional regulation.

## 14 Potential Applications of IAA in Rheumatoid Arthritis Treatment

Rheumatoid arthritis is an autoimmune disease characterized by chronic inflammation of synovial joints, leading to cartilage and bone damage. The pathological processes contributing to joint deformity and damage include inflammation, angiogenesis (formation of new blood vessels), and osteoclastogenesis (bone resorption) [91]. Recent attention has been drawn to indole derivatives produced by gut microbiota, as their concentrations in the synovial fluid of individuals with rheumatoid arthritis are lower than those with osteoarthritis [92]. However, their precise impact on disease progression remains under investigation. A study discovered the effects of two MDTMs, indole‐3‐aldehyde and IAA, on the processes associated with arthritis. Comparative analyses in mouse cell models revealed distinct functional differences despite their structural similarities. Indole‐3‐aldehyde demonstrated anti‐inflammatory activity along with proosteoclastogenic and proangiogenic effects. Interestingly, these effects appear to involve mechanisms independent of the AhR. On the other hand, IAA exhibited AhR‐dependent antiangiogenic activity [93]. The conflicting effects of these indole derivatives on angiogenesis highlight their complex roles in the disease. Indole‐3‐aldehyde's anti‐inflammatory properties may help manage moderate inflammation triggered by subclinical infections or injuries, thereby protecting against inflammatory arthritis. However, its proangiogenic and proosteoclastogenic properties could exacerbate arthritis pathology if its levels become excessively elevated. In contrast, IAA's antiangiogenic activity may counterbalance the proangiogenic effects of indole‐3‐aldehyde in disease contexts [93].

## 15 Microbiota‐Derived 3‐IAA Protects Against Sepsis‐Associated Encephalopathy

Sepsis‐associated encephalopathy (SAE) is a widespread neurological complication in sepsis. This arises from neuroinflammation, cerebral perfusion issues, blood–brain barrier (BBB) dysfunction, and neurotransmitter dysregulation [94]. Management of SAE is limited to infection control and symptomatic treatment for delirium and seizures. Gut MDTMs have mainly been considered for their anti‐inflammatory and antioxidative roles, with limited exploration of their potential in SAE [62, 95]. Suggestions suggest that MDTMs can cross the BBB and reduce CNS inflammation [96, 97]. In septic patients, lower levels of IAA were observed in those with SAE, correlating negatively with neurocognitive dysfunction and sepsis mortality risk. Huang et al. found that IAA mitigated SAE through survival and behavioral studies and cytokine analysis in cortical brain tissue [97]. However, the inflammatory status of other brain regions remains unexplored and needs further research.

Sepsis‐induced microglial activation leads to oxidative damage, BBB disruption, and elevated pro‐inflammatory cytokines such as TNF‐α, IL‐1, and IL‐6, contributing to persistent neuroinflammation and long‐term cognitive decline [98, 99]. Studies demonstrate that microglial activation plays a pivotal role in SAE progression. Huang et al. reported that IAA inhibited microglial activation and improved survival in septic mice [97]. The fecal IAA level was reduced in patients with sepsis‐associated delirium (SAD), a manifestation of SAE. Decreased fecal IAA level in SAD patients was associated with worsening clinical outcomes. A lower survival rate in post‐CLP modeling was observed in the mice transplanted with the fecal bacteria from the SAD patients compared to those transplanted with fecal bacteria from the NSAD. Oral administration of IAA ameliorated CNS inflammation and alleviated SAE‐like symptoms in septic mice through enhancing AhR activity. However, preemptive microglial depletion negated IAA's therapeutic effects, representing microglia as important mediators between gut microbiota and CNS [100]. The AhR activity induced by CLP was augmented by IAA in vivo and further enhanced in microglia co‐treated with IAA and LPS. It suggests that the beneficial effect of IAA against SAE may involve a noncanonical AhR pathway [97]. Moreover, by short‐term TLR4 stimulation, IAA may act as a TLR4 ligand, suppressing TNF‐α expression [101]. More investigations are needed to clarify the pathways through which IAA protects against SAE.

## 16 Conclusions

IAA has a significant therapeutic potential across a range of diseases, while also highlighting the complexities of its effects in various circumstances. In CKD, higher IAA levels contribute to dysfunction of CV and damage to the liver [43, 47, 51, 54]; so far they also exhibit protective effects in NAFLD [60]. Furthermore, IAA has revealed promise in improving inflammation, modulating immune responses, and stabilizing cellular barriers in lung conditions like ARDS, PF, and COPD [12, 23, 71, 72, 76]. In the case of cancer therapy, IAA's combined effect with chemotherapy agents and its effect on reactive oxygen species and autophagy point to its potential in improving treatment results for PDAC [71, 72, 84]. *A.* *radioresistens‐derived* IAA inhibits cellular proliferation, ISC turnover, and tumorigenesis in mouse models of CRC, which highlights IAA's potential therapeutic role in the treatment of CRC [82].

**FIGURE 3:**
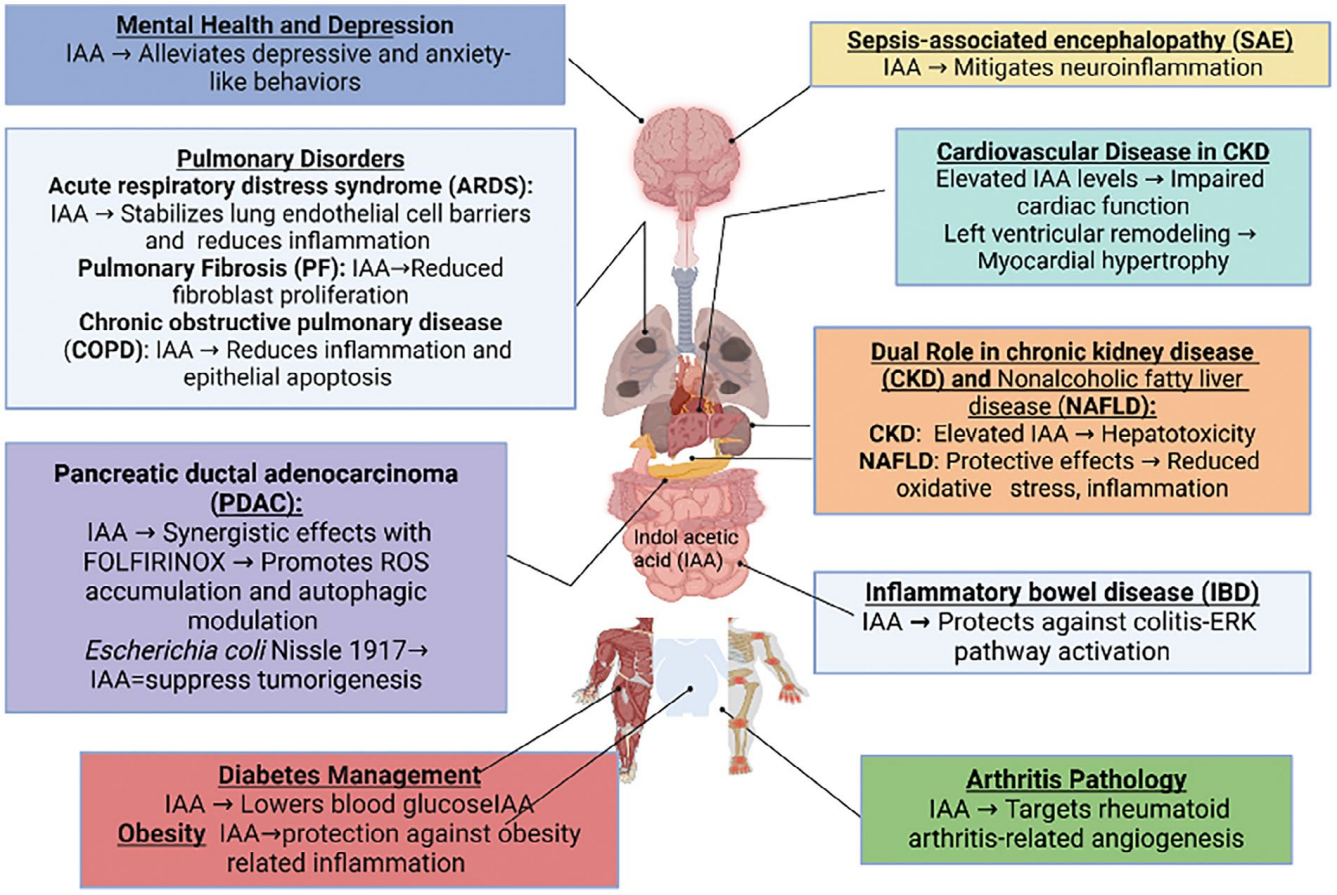
The multifaceted roles of IAA in human diseases. The diverse effects of IAA on various human organs, highlighting its dual role depending on the pathological context. In some conditions, IAA exhibits a protective or promotive effect by enhancing tissue repair, modulating immune responses, or supporting cellular growth. On the other hand, IAA may accelerate disease progression, contribute to tissue damage, or disrupt normal physiological functions. This variability underscores the complexity of IAA's role in human health and disease. The figure was created with biorender.com.

IAA's effect extends to the CNS, where it modulates gut–brain interactions to alleviate anxiety‐like and depressive behaviors, and in metabolic diseases (i.e., obesity and diabetes), where it presents a promising alternative due to its antioxidant potential [17, 65, 69]. IAA also displays therapeutic promise in managing rheumatoid arthritis through its activity of antiangiogenic [93]. In SAE, IAA also plays an important role in promoting cognitive recovery and mitigating neuroinflammation [97]. Briefly, while IAA demonstrates diverse therapeutic effects, its dual nature underscores the importance of context in its application (Figure 3).

## 17 Future Recommendations

Future research should focus on elucidating the mechanisms through which IAA influences its role in CV, liver, pulmonary, and neurological diseases in various physiological and pathological processes. The studies should explore the optimal dosing strategies, safety profiles, and therapeutic potential of IAA in chronic conditions like CKD, IBD, NAFLD, PDAC, and CRC. Moreover, investigating IAA's effects on the gut–brain axis, its impact on immune responses, and its modulation of oxidative stress could offer new insights into its role in mental health, diabetes, and sepsis. Ultimately, this research will pave the way for targeted therapeutic approaches using IAA and its derivatives.

## Author Contributions

Nargis Shaheen drafted the article, the table, and the figures. Jiaxing Miao and Boyu Xia collected the related papers and conducted citation verification. Yutong Zhao and Jing Zhao designed and revised the article. All authors read and approved the final version of the review.

## Conflicts of Interest

The authors declare no conflicts of interest.

## Data Availability

The authors have nothing to report.
